# Self-assembly formation of hollow Ni-Fe-O nanocage architectures by metal-organic frameworks with high-performance lithium storage

**DOI:** 10.1038/srep13310

**Published:** 2015-09-08

**Authors:** Hong Guo, Tingting Li, Weiwei Chen, Lixiang Liu, Jinli Qiao, Jiujun Zhang

**Affiliations:** 1 School of Chemistry Science and Engineering, Yunnan University, Kunming 650091,Yunnan, China; 2 College of Environmental Science and Engineering, Donghua University, Shanghai 201620, China; 3 NRC Energy, Mining & Environment Portfolio, National Research Council Canada, Vancouver, BC V6T 1W5, Canada

## Abstract

A hollow hybrid Ni-Fe-O nanomaterial (NiFe_2_O_4_) is synthesized using a precursor of metal-organic frameworks through a simple and cost-effective method. The unique hollow nanocage structures shorten the length of Li-ion diffusion. The hollow structure offers a sufficient void space, which sufficiently alleviates the mechanical stress caused by volume change. Besides, the hybrid elements allow the volume change to take place in a stepwise manner during electrochemical cycle. And thus, the hierarchical hollow NiFe_2_O_4_ nanocage electrode exhibits extraordinary electrochemical performance. The stable cyclic performance is obtained for all rates from 1 C to 10 C. Even when the current reaches 10 C, the capacity can also arrive at 652 mAhg^−1^. Subsequently, a specific capacity of ca. 975 mAhg^−1^ is recovered when the current rate reduces back to 1 C after 200 cycles. This strategy that derived from NMOFs may shed light on a new route for large-scale synthesis of hollow porous hybrid nanocages for energy storage, environmental remediation and other novel applications.

Currently the efforts in developing advanced materials for energy and environmental applications have become even more intensive driven by the urgency of increasing energy efficiency and environment protection^1^,^2^,^3^,^4^. In particular, electrode materials with precisely constructed nano-structured architectures have attracted considerable attention due to their potential applications in high-performing electrochemical energy devices such as batteries, fuel cells and supercapacitors. With respect to this, many efforts have been focused on the research to control the size, morphology, composition, crystallinity and building blocks of the materials with hollow structures, which have well-defined interior voids, high specific surface area, low density, accommodate volume change without pulverizing compared with that of solid counterparts of the same sized ones^5^,^6^,^7^,^8^,^9^. For instance, TiO_2_ based hollow materials with different sizes and morphologies could show some enhanced electrochemical properties^7^,^10^,^11^. Our previously prepared hollow cage-bell Ag@TiO_2_ materials have demonstrated excellent lithium-ion storage capability^12^. Zhou *et al*.^13^ and Wang *et al*.^14^ synthesized hollow microcubes of metal sulfides for batteries, and the resulted electrodes gave some enhanced lithium storage capability. Most recently, several novel hybrid hollow materials containing multiple layers of shells or multiple compositions were explored and showed desirable and unique properties and also maximized structural advantages when compared to single component hollow structured materials^15^,^16^,^17^,^18^,^19^,^20^. However, most of the current available hollow materials were synthesized by multistep template routes, which could have some challenges such as difficulty in template fabrication, low product yields, and multistep and costly operations. Therefore, some new and effective approaches to rationally fabricate hierarchical hollow hybrid nanomaterials are necessary in exploring these materials through a more simple, facile and environment-friendly process.

Regarding the templates used for hollow structured electrode materials, nanostructured metal-organic frameworks (NMOFs)^21^,^22^,^23^, a new class of organic-inorganic hybrid functional materials with high porosity and large surface area, seem to be very attractive, which can allow to be easy tuned for selection of different metal ions and organic bridging ligands^10^,^11^. They have been proved to be effective templates for preparing hollow-structured transition metal oxides by thermal decomposition. This is because the porosity and long-range ordering of NMOFs can offer a fast and convenient access for incoming and leaving small molecules and ions in the transformation process^11^. In this regard, when compared to other templates/precursors, NMOFs can offer distinct advantages due to their unique structure and porosity and/or hollow structures. The advantage of NMFOs as templates gives them a tremendous application opportunity in developing a series of highly tailorable functional nanomaterials. For example, these NMOFs were used to synthesize hollow metal oxides, which exhibited a much enhanced electrochemical performance when evaluated as anode materials for Lithium-ion batteries (LIBs)^24^. Low *et al*.^25^ prepared Fe_2_O_3_ microboxes with hierarchically structured shells simply via annealing Prussian blue (PB) microcubes in air. Cho *et al*.^26^ reported a spindle-like porous α-Fe_2_O_3_ prepared from a typical iron-based NMOF template (MIL-88-Fe). Most recently, some nonspherical hollow-structured porous shell materials were synthesized by our group and others^24^,^27^.

In this work, a hybrid nanostructured NiFe_2_O_4_ with a hollow nanocage structure is synthesized to validate our proposed strategy in preparing hollow porous nanocages using NMOFs as a template (Fig. 1). The advantages in choosing such a Fe- and Ni- containing material is due to the low cost, non-toxicity and natural abundance of these two transition metal oxides as well as their high capacities when used as anode materials for LIBs^24^,^25^,^26^,^27^,^28^. However, although the Ni and Fe oxides-based LIB electrodes can give high capacity, the pulverization of the electrode caused by huge volume change during the process of electrochemical reaction could lead to a rapid capacity degradation and poor cycling stability. When using a hybrid nanostructured NiFe_2_O_4_ prepared in this work, this issue could be significantly overcome, allowing the electrochemical reaction to proceed in a hybrid matrix of distinct material systems and resulting in an effective control of the electrode volume changes through facilitating the step-wise manner rather than at a certain fixed potential. Moreover, the coupling of these two metal species could render the NiFe_2_O_4_ with rich redox reactions and then improve electronic conductivity. As a result, the ternary NiFe_2_O_4_ prepared exhibits a higher electrical conductivity and an improved electrochemical activity when compared to binary metal oxides Fe_2_O_3_ and NiO^2^,^24^. Furthermore, this hollow nanocage structured NiFe_2_O_4_ could allow small-sized Lithium ion (Li^+^) to fast diffusion for reversible lithium ion storage, resulting in a good toleration to volume change during cycling^12^,^13^,^14^. This paper reports the geometric configuration of hierarchical hollow NiFe_2_O_4_ nanocages synthesized using nano metal-organic frameworks and its associated high electrochemical performance when used as the anode materials for LIBs.

## Experimental

### Synthesis of hollow porous NiFe_2_O_4_ nanocages

All chemicals used in this work were analytical grade and used without further purification. For a typical synthetic experiment, Solution A was synthesized by dissolving 0.187 g of Ni(CH_3_COO)_2_ and 0.993 g of sodium citrate in 100 mL of distilled water with stirring. Solution B was prepared by adding 1.32 g of K_3_[Fe(CN)_6_] into 100 mL of aqueous solution of PVP K30 (4 g) with stirring. Then Solution A and Solution B were mixed together under stirring. After 20 minutes of stirring, an eggplant turbid liquid was formed and then aged for 24 hours for forming a precipitate. Then the precipitate was treated by several centrifuge-wash cycles with distilled water and ethanol, and then dried at room temperature for 10 hours. The collected powder of hollow porous NiFe_2_O_4_ nanocages was annealed in Air at 350 °C for 2 hours at a heating rate of 1 °C min^−1^ to obtain the product.

### Material characterization

X-ray diffraction (XRD) was used to identity the phase composition of synthesized samples over the 2*θ* range from 20° to 90° using a Rigaku D/max-A diffractometer with Co Kα radiation. A Fourier transform infrared spectroscope (FTIR, Themo Nicolet 670FT-IR) was used for recording the FTIR spectra of the sample ranged from 500 to 4000 cm^−1^. The microstructural characteristics of NiFe_2_O_4_ nanocages were recorded by high-resolution transmission electron microscope (HR-TEM, JEOL JEM-2010) working at 200 kV accelerating voltage, and the lattice structure was identified by selected area electron diffraction (SAED) technique.

### Electrochemical characterization

For electrochemical performance evaluation, half-cell measurements were performed. For preparation of working electrode containing NiFe_2_O_4_ nanocages, acetylene black powder and polyvinylidene fluoride (PVDF) were used as conductive additive and binder, respectively. In the preparation, the synthesized NiFe_2_O_4_ nanocages were mixed with acetylene black and PVDF dissolved in N-methyl-pyrrolidinone in a weight ratio of 85:10:5 to form slurry, which was then painted on a copper foil to form the working electrode. This copper foil was served as both the material support and current collector. After solvent evaporation, the electrode was pressed and dried at 120 ^o^C under vacuum for 48 hours. The electrochemical cells were assembled in a argon filled glove-box. Metallic lithium foil was used as the counter electrode. The electrolyte was 1M LiPF_6_ in a mixture of ethylene carbonate (EC) and dimethyl carbonate (DMC) (1:1 in vol. ratio). Cycling tests were carried out at different current densities (1c = 914 mAg^−1^), in the voltage range of 0.01–1.5 V versus Li/Li^+^ by using a Land 2100A tester.

## Results and Discussion

The crystallographic structure and phase purity of the precursor and as-synthesized hollow porous NiFe_2_O_4_ nanocages are analyzed by XRD, as shown in Fig. 2a,b. From Fig. 2a and compared the spectrum with standard one (JCPDS card no. 46–0908), it can be seen that the precursor Ni_2_Fe(CN)_6_ does not contain impurity. All the diffraction peaks of final products, shown in Fig. 2b, can be indexed to the monoclinic phase of NiFe_2_O_4_ (JCPDS card no. 10–0325) without impurity peaks, indicating a complete thermal conversion of the NMOF precursors into NiFe_2_O_4_ nanostructures. The FTIR spectrum images of the prepared hollow structured NiFe_2_O_4_ nanocage sample and its Ni_2_Fe(CN)_6_ precursor are shown in Fig. 3. The broad absorption peaks centered at ca. 3391 to 2357 cm^−1^ can be assigned to the stretching vibrations of the -OH group of absorbed water molecules and absorption of CO_2_ in the air. According to the spectrum of Ni_2_Fe(CN)_6_, the peaks from 1654 to 1601 cm^−1^ can be assigned to the bending vibrations of the water molecules; the spike of 2108 cm^−1^ is assigned to the stretching vibrations of the cyano group of precursor. These peaks are all disappeared in the spectrum of synthesized NiFe_2_O_4_ samples, indicating that these groups have decomposed after the calcinations. The strongest broad peaks in the range of 992 to 500 cm^−1^ are contributed from the bands of metal-oxide^29^.

Figure 4a,b show the SEM and TEM images of the Ni_2_Fe(CN)_6_ precursors. It is clear that the precursors are solid submicro-cubes with an average diameter of ca.100 nm according to Fig. 4a. A smooth surface can be seen on these solid submicro-cubes, as in Fig. 4b. The hollow porous morphology of NiFe_2_O_4_ nanocage sample is also characterized by SEM, TEM and HR-TEM, as illustrated in Fig. 4c–f. After calcination of Ni_2_Fe(CN)_6_ precursors at 350 °C for 4 hours, a fluffy black powder contains uniformly distributed nanocubes with a shrunk size as in Fig. 4c. It is interesting to find that the NiFe_2_O_4_ sample from NMOFs is not a solid box but a visible hollow porous interior structure with an average diameter of ca. 80 nm, as evidenced by the partial broken shell vividly, as shown in Fig. 4d. Particularly, a typical structure with well-defined interior and thin shell can also be detected. The size of as-obtained nanocages is much smaller than the previously reported porous nanostructures derived by NMOFs recently^25^. It is believed that the hollow porous structure of these particles might be induced by a rapid mass-transport from core to shell during the calcinations. The surface of the synthesized NiFe_2_O_4_ powder is made up from nano-sized small particles of ca. 3–9 nm, which is in a good agreement with XRD results. The selected-area electron diffraction (SAED) pattern (Fig. 4e,f) of one typical particle reveals the diffraction rings can be indexed to (2 2 0), (3 1 1), and (4 0 0) diffraction of face-centered cubic NiO, respectively. The lattice fringe with a lattice spacing (0.251 nm) can be seen clearly, agreeing with NiFe_2_O_4_ (3 1 1) plane spacing (Fig. 4f).

The N_2_ adsorption/desorption isotherms and the pore size distribution of obtained hollow porous NiFe_2_O_4_ nanocages from NMOFs are shown in Fig. 5. The isotherms are typical Type IV, which are the characteristic isotherm of mesoporous materials. The pore size distribution data indicates that the pore diameter distribution is in the range of 3–8 nm. The BET surface area of the sample is 260.9 m^2^ g^−1^. It can be seen that the specific surface area of NiFe_2_O_4_ is significantly higher than most of the previous reported TMOs microsphere products^24^,^25^,^26^. The single-point total volume of pores at P/P_0_ = 0.975 is 0.438 cm^3^ g^−1^. These data indicate that the prepared samples have a loose mesoporous structure. This structure is believed to have beneficial effect on buffering the volume changes of hollow porous NiFe_2_O_4_ nanocage electrodes during electrochemical reaction.

**Figure 1:**
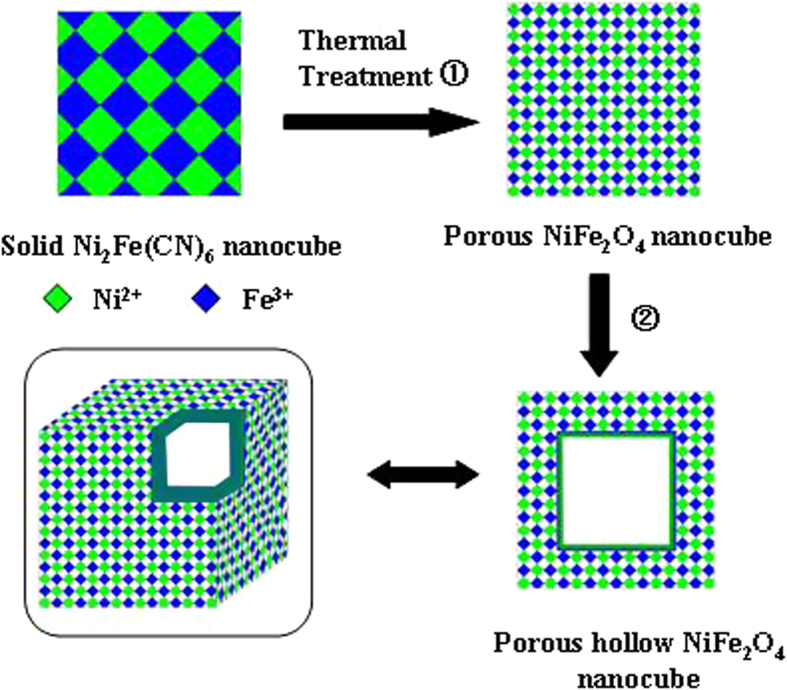
Representative illustration of the formation of hollow NiFe_2_O_4_ nanocages for MOFs. (This figure is drawn by T.T.L.).

**Figure 2:**
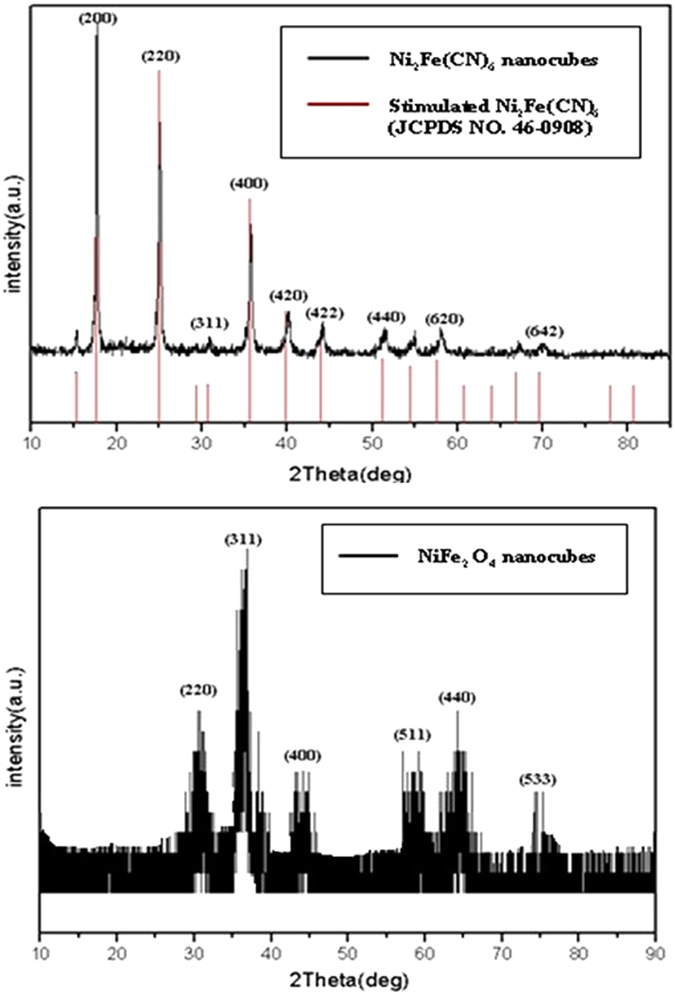
XRD pattern of hollow NiFe_2_O_4_ nanocages (**b**) and its precursor (**a**).

**Figure 3:**
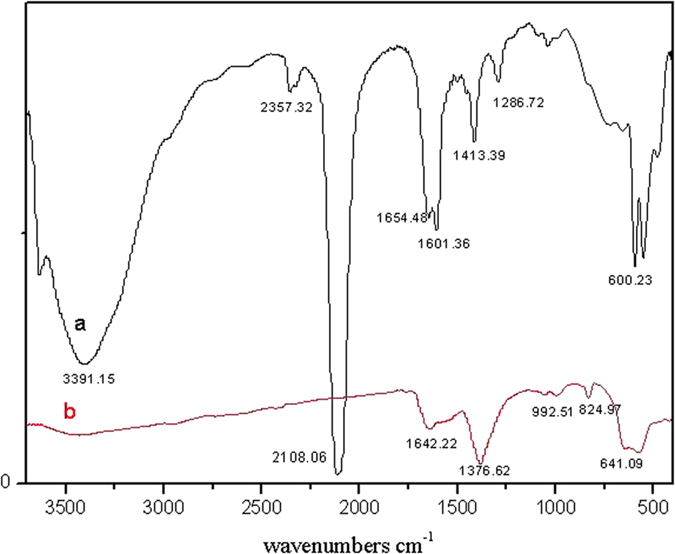
FTIR spectra of hollow NiFe_2_O_4_ nanocages (**b**) and its precursor (**a**).

**Figure 4:**
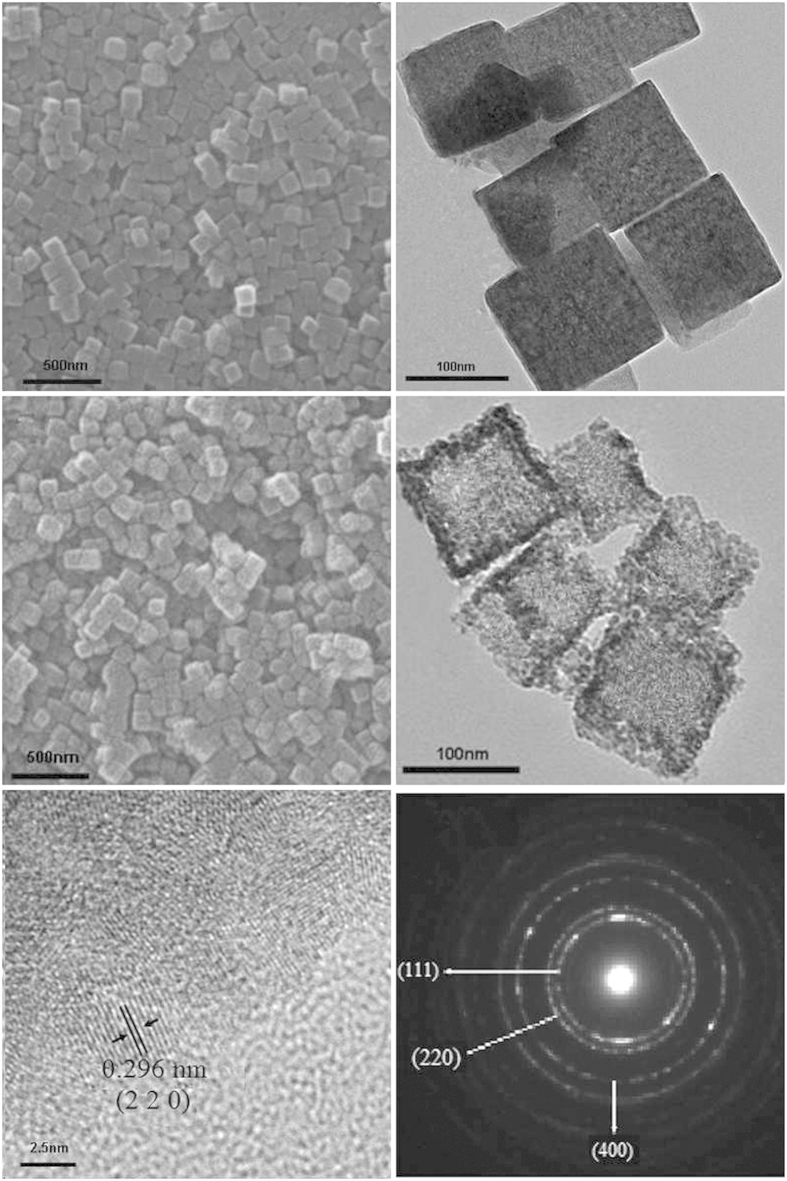
SEM (**a**) image and TEM (**b**) image of prepared Ni_2_[Fe(CN)_6_] nanocages (**a**) and hollow NiFe_2_O_4_ nanocages. SEM images (**c**), TEM (**d**) images, HRTEM micrographs (**e**) and selected area electron diffraction (SAED) (**f**) of as-synthesized hollow NiFe_2_O_4_ nanocages yielded by calcination at 350 °C.

**Figure 5:**
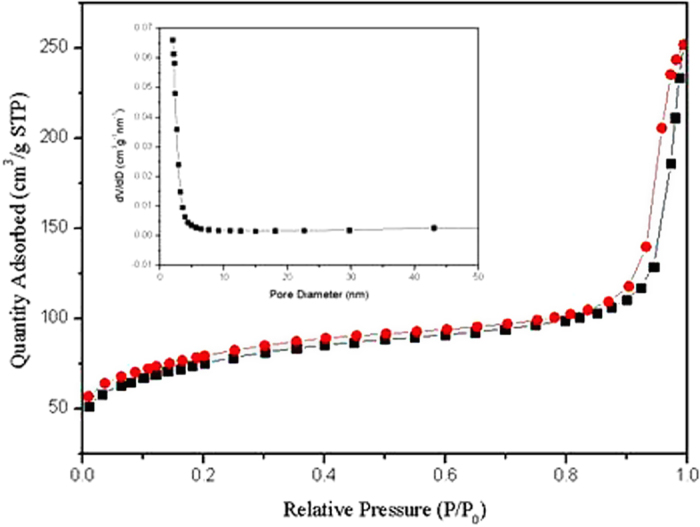
N_2_ adsorption/desorption isotherm (77 K) curve for hollow NiFe_2_O_4_ nanocages (350 °C). Inset: The pore-size distribution of the samples.

The formation mechanism can be described as schematically illustrated in Fig. 1 by the following Equation (1). When Ni_2_Fe(CN)_6_ is converted into NiFe_2_O_4_, the carbon and nitrogen in CN^–^ are oxidized into gases and escaped.


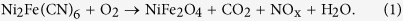


**Figure 6:**
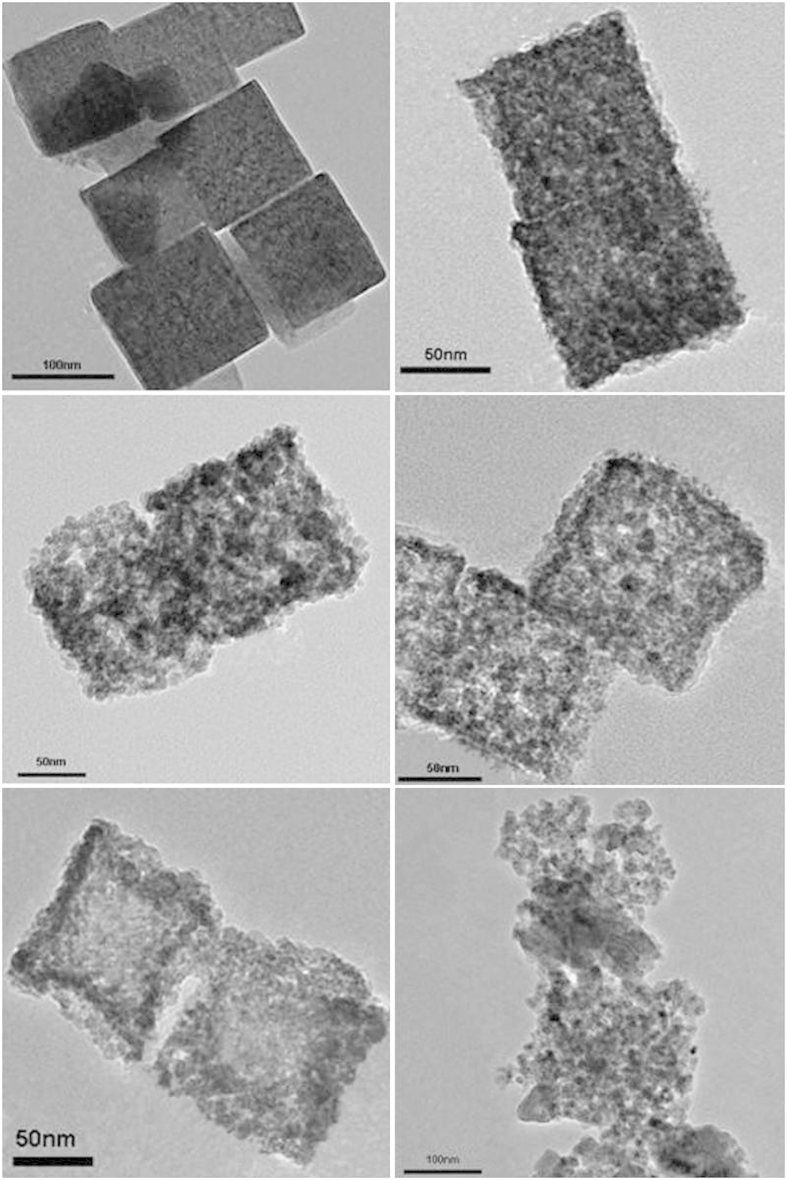
TEM images of porous NiFe_2_O_4_ nanocages obtained at (**a**) 150 °C, (**b**) 200 °C, (**c**) 250 °C, (**d**) 300 °C, (**e**) 350 °C, and (**f**) 400 °C for 4 h.

The formation mechanism can be further investigated through a series of experiments as shown in Fig. 6, which presents the TEM images of NiFe_2_O_4_ obtained after calcination at 150 °C, 200 °C, 250 °C, 300 °C, 350 °C and 400 °C for 4 hours, respectively. It can be seen that only solid nanocages with a smooth surface can be formed after calcination at 150 °C (Fig. 6a). When the reaction temperature is increased to 200 °C, the sample surface becomes a little coarse and shows a porous structure (Fig. 6b). With continuously increasing the temperature up to 250 °C, the sample surface become even more coarser, and the hollow configuration is appearing gradually (Fig. 6c). It can be significantly noticed that the porous nanocage can be transformed into hollow one while the temperature is increased to 300 °C (Fig. 6d). With further increasing the temperature, the interior of the nanocage structure is broken and nanocage becomes a small nano particle with sizes of ca. 4–8 nm, as shown in Fig. 6e,f which are corresponding to those at 350 °C and 400 °C, respectively. According to these observations, it can be speculated that the formation of unique hollow porous NiFe_2_O_4_ nanocages go through the transformation from solid to hollow ones, which may be induced by the quick evolution of CO_2_ and NO_x_ gases during the thermal decomposition process. It can be stated here that our strategy, as shown in Fig. 1, can provide a novel procedure and simple route to prepare hierarchical hollow porous nanocages from NMOFs with higher BET surface and larger quantity.

**Figure 7:**
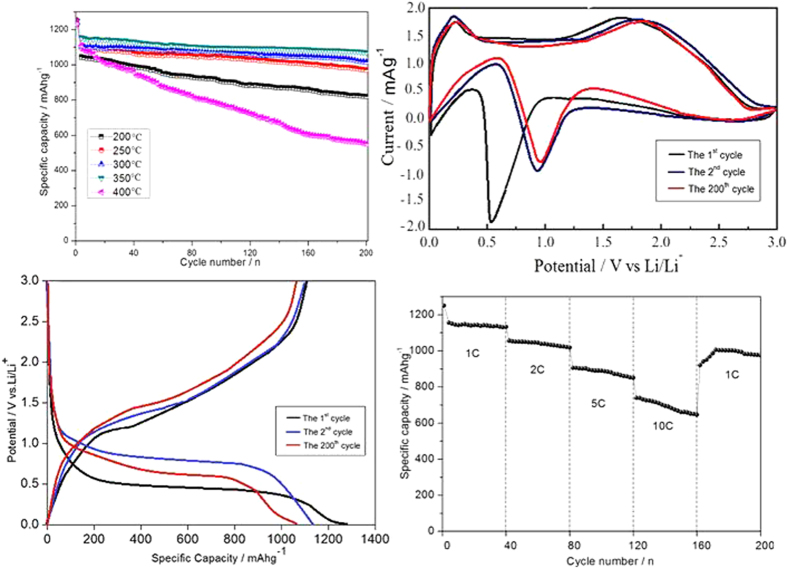
Electrochemical performance of prepared hierarchical porous hollow NiFe_2_O_4_ nanocage electrode: (**a**) cycling performance of CoFe_2_O_4_ materials at different temperatures from 200 °C to 400 °C at constant current density of 1 C; (**b**) the cycle of CV curve with a scan rate of 0.05 mVs^−1^; (**c**) charge/discharge curves of CoFe_2_O_4_ (350 °C) electrode for the 1^st^, 2^nd^, and 200^th^ cycle at current density of 1 C; (**c**) the first cycle CV curve with a scan rate of 0.05 mVs^−1^; (**d**) rate capability of NiFe_2_O_4_ electrode from 1 C to 20 C for 200 cycles. Electrode potential range of 0.01–3.0 V vs. Li/Li^+^.

The electrochemical performance of the prepared hollow NiFe_2_O_4_ nanocages as anode material of Li-ion batteries was also tested. As shown in Fig. 7a, the increase in cycling stability and capacity with increasing temperature from 200 °C to 400 °C is mainly attributed to the formation of hollow porous structure. The capacity of the sample at 350 °C remains a stable value as high as 1071 mAhg^−1^ after 200 cycles. However, its capacity fades drastically from 1242 to 552 mAh g^−1^ after 200 cycles when the temperature is increased to 400 °C, which is caused by the collapse of hollow structure in the process of calcination at high temperature as evidenced in Fig. 6. The CV and charge/discharge curves of hierarchical hollow NiFe_2_O_4_ nanocage (350 °C) electrode are shown in Fig. 7b,c, respectively. In the first scan of CV, two cathodic peaks can be observed at 0.54 and 1.51 V, corresponding to the conversion reactions of Fe^3+^ and Ni^2+^ to their metallic states and the formation of Li_2_O^30^. The broad anodic peaks can be ascribed to the oxidation reactions of metallic Fe and Ni. The reactions of NiFe_2_O_4_ with Li can be written as Equations (2) and (3)^31^:


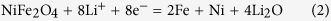



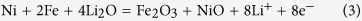


**Figure 8:**
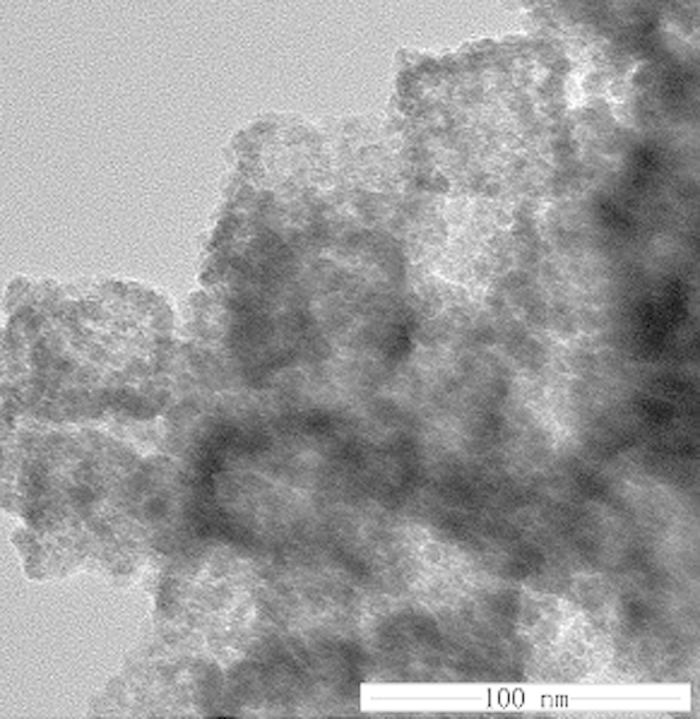
TEM image of hollow structured NiFe_2_O_4_ nanocage electrodes after 200 cycles at 1C.

Similar to the simple oxide Fe_2_O_3_ and NiO, the mixed oxide NiFe_2_O_4_ stores Li through reversible formation and decomposition of Li_2_O. In the second scan, the reduction peaks are shifted to 0.94 and 1.56 V, respectively. The asymmetric nature of the plots suggests that the conversion reactions are only partially reversible and complete structural recovery to NiFe_2_O_4_ cannot occur^32^. As shown in Fig. 7c, the 1^st^ discharge (Li^+^ insertion) and charge (Li^+^ extraction) curve at a current density of 1 C in the voltage window of 0.01–3 V (Fig. 7c) shows a wide, steady discharging plateau at ca. 0.65 V, followed by a gradual voltage decrease. The initial discharge and charge capacities are 1245 mAhg^−1^ and 1152 mAhg^−1^, respectively. The initial capacity loss should be attributed to the formation of solid electrolyte interphase (SEI) and the reduction of metal oxide to metal with Li_2_O formation. These results are consistent with CV analysis. From the second cycle onwards, the long potential plateau is replaced by a long slope between 1.2 and 0.73 V. After 200 cycles, the capacity can also be kept at 1071 mAhg^−1^, showing the excellent reversibility of electrode. To further investigate electrochemical performance, Fig. 7d shows the discharge capacities of NiFe_2_O_4_ electrode at different charging rates from 1 C to 10 C with 40 cycles. It can be seen that the stable cyclic performance can be obtained at all rates. Even when the current reaches 10 C, the capacity can also maintain at 652 mAh g^−1^. Subsequently, a specific capacity of ca. 975 mAhg^−1^ is recovered when the current rate reduces back to 1 C after 200 cycles. The overall rate performance demonstrates the high capacities in both low and high current rates of the prepared NiFe_2_O_4_ nanocage electrode. In Fig. 8, the TEM of NiFe_2_O_4_ electrode after 200 cycles at a current rate of 1 C reveals that the structure of this anode material can be retained well without breakage in the process of charge-discharge. Compared with the previously reported TMOs materials^24^,^33^,^34^,^35^,^36^, the synthesized NiFe_2_O_4_ material in this work exhibits an enhanced electrochemical performance with our novel strategy. The unique hollow nanocage structures can shorten the length of Li-ion diffusion, which is beneficial to the rate performance. Furthermore, the hollow structure can offer a sufficient void space, sufficiently alleviating the mechanical stress caused by the volume change. Besides, the hybrid elements allow the volume change to take place in a stepwise manner during electrochemical cycle. As a result, the hierarchical hollow NiFe_2_O_4_ nanocage electrode can exhibit an extraordinary high electrochemical performance.

## Conclusions

In summary, material with a structure of hollow porous NiFe_2_O_4_ nanocage from NMOFs is successfully synthesized by a facile and fast benign procedure. When it is used as an anode material for Lithium-ion battery, a stable reversible capacity can be retained at 1017 mAhg^−1^ after 200 cycles with an excellent rate performance. This novel synthetic strategy may shed light on a novel avenue for the effective synthesis of hollow porous hybrid nanocage materials derived from NMOFs for energy storage, sensor, catalyst, environmental remediation and other new applications.

## Additional Information

**How to cite this article**: Guo, H. *et al*. Self-assembly formation of hollow Ni-Fe-O nanocage architectures by metal-organic frameworks with high-performance lithium storage. *Sci. Rep*. **5**, 13310; doi: 10.1038/srep13310 (2015).

## References

[b1] Liu J. . Monodisperse yolk–shell nanoparticles with a hierarchical porous structure for delivery vehicles and nanoreactors. Angew. Chem. Int. Ed. 122, 5101–5105 (2010). 10.1002/anie.201001252 20512832

[b2] Wang B., Chen J. S., Wu H. B., Wang Z. & Lou X. W. Quasiemulsion-templated formation of α-Fe_2_O_3_ hollow spheres with enhanced lithium storage properties. J. Am. Chem. Soc. 133, 17146–17148 (2011). 21977903 10.1021/ja208346s

[b3] Bian Z. . Multitemplates for the hierarchical synthesis of diverse inorganic materials. J. Am. Chem. Soc. 134, 2325–2331 (2012). 22239167 10.1021/ja210270m

[b4] Lou X. W., Li C. M. & Archer L. A. Designed synthesis of coaxial SnO_2_@ carbon hollow nanospheres for highly reversible lithium storage. Adv. Mater. 21, 2536–2539 (2009).

[b5] Guo H. . Core-shell TiO_2_ microsphere with enhanced photocatalytic activity and improved lithium storage. J. Solid State Chem. 201, 137–143 (2013).

[b6] Yin Y. . Formation of hollow nanocrystals through the nanoscale Kirkendall effect. Science 304, 711–714 (2004). 15118156 10.1126/science.1096566

[b7] Liu L. . Hollow NiO nanotubes synthesized by bio-templates as the high performance anode materials of lithium-ion batteries. Electrochim. Acta 114, 42–47 (2013).

[b8] Zhang Q., Lee I., Joo J. B., Zaera F. & Yin Y. Core–shell nanostructured catalysts. Acc. Chem. Res. 46, 1816–1824 (2012). 23268644 10.1021/ar300230s

[b9] Yao Y. . Interconnected silicon hollow nanospheres for lithium-ion battery anodes with long cycle life. Nano lett. 11, 2949–2954 (2011). 21668030 10.1021/nl201470j

[b10] Guo H. . Template-free fabrication of hollow NiO–Carbon hybrid nanoparticle aggregates with improved lithium storage. Part. Part. Syst. Char. 31, 374–381 (2014).

[b11] Lai X. . General synthesis and gas-sensing properties of multiple-shell metal oxide hollow microspheres. Angew. Chem. Int. Ed. 123, 2790–2793 (2011). 10.1002/anie.201004900 21387478

[b12] Guo H. . Shape-controlled synthesis of Ag@ TiO_2_ cage-bell hybrid structure with enhanced photocatalytic activity and superior lithium storage. Green Chem. 15, 2810–2816 (2013).

[b13] Zhou L., Zhao D. & Lou X. W. Double-shelled CoMn_2_O_4_ hollow microcubes as high-capacity anodes for lithium-ion batteries. Adv. Mater. 24, 745–748 (2012). 22213232 10.1002/adma.201104407

[b14] Wang Z. Wang Z., Liu W., Xiao W. & Lou X. W. D. Amorphous CoSnO_3_@C nanoboxes with superior Lithium storage capability. Energy Environ. Sci. 6, 87–91 (2013).

[b15] Guo H. . Morphology-controlled synthesis of cage-bell Pd@ CeO_2_ structured nanoparticle aggregates as catalysts for the low-temperature oxidation of CO. J. Mater. Chem. A 1, 7494–7499 (2013).

[b16] Wu C. . Facile strategy for synthesis of silica/polymer hybrid hollow nanoparticles with channels. Langmuir 26, 18503–18507 (2010). 21062000 10.1021/la103629v

[b17] Wang Y., Su F., Lee J. Y. & Zhao X. S. Crystalline carbon hollow spheres, crystalline carbon-SnO_2_ hollow spheres, and crystalline SnO_2_ hollow spheres: synthesis and performance in reversible Li-ion storage. Chem. Mater. 18, 1347–1353 (2006).

[b18] Deng J. . Sandwich-stacked SnO_2_/Cu hybrid nanosheets as multichannel anodes for lithium ion batteries. Acs Nano 7, 6948–6954 (2013). 23879640 10.1021/nn402164q

[b19] Nelson K. & Deng Y. Enhanced light scattering from hollow polycrystalline TiO_2_ particles in a cellulose matrix. Langmuir 24, 975–982 (2008). 18179269 10.1021/la702582u

[b20] Wang D. P. & Zeng H. C. Multifunctional roles of TiO_2_ nanoparticles for architecture of complex core-shells and hollow spheres of SiO_2_-TiO_2_−Polyaniline System. Chemistry of Materials 21, 4811–4823 (2009).

[b21] Wu Y. F., Han J. Y., Xue P., Xu R. & Kang Y. J. Nano metal-organic framework (NMOF)-based strategies for multiplexed micro RNA detection in solution and living cancer cells. Nanoscale 7, 1753–1759 (2015). 25514895 10.1039/c4nr05447d

[b22] Jana J. A., Jorge G. & Freek K. Metal-organic frameworks as scaffolds for the encapsulation of active species: state of the art and future perspectives. J. Mater. Chem. 22, 10102–10118 (2012).

[b23] Falcaro P. . MOF positioning technology and device fabrication. Chem. Soc. Rev. 43, 5513–5560 (2014). 24802634 10.1039/c4cs00089g

[b24] Hu L. & Chen Q. Hollow/porous nanostructures derived from nanoscale metal–organic frameworks towards high performance anodes for lithium-ion batteries. Nanoscale 6, 1236–1257 (2014). 24356788 10.1039/c3nr05192g

[b25] Zhang L., Wu H. B. & Lou X. W. Metal–organic-frameworks-derived general formation of hollow structures with high complexity. J. Am. Chem. Soc. 135, 10664–10672 (2013). 23805894 10.1021/ja401727n

[b26] Xu X., Cao R., Jeong S. & Cho J. Spindle-like mesoporous α-Fe_2_O_3_ anode material prepared from MOF template for high-rate lithium batteries. Nano lett. 12, 4988–4991 (2012). 22881989 10.1021/nl302618s

[b27] Guo H. . Accurate hierarchical control of hollow crossed NiCo_2_O_4_ nanocubes for superior lithium storage. Nanoscale 6, 5491–5497 (2014). 24728284 10.1039/c4nr00930d

[b28] Hu M. . Synthesis of superparamagnetic nanoporous iron oxide particles with hollow interiors by using Prussian blue coordination polymers. Chem. Mater. 24, 2698–2707 (2012).

[b29] Griffiths P. R., Haseth J. A. D. Fourier Transform Infrared Spectrometry, John Wiley & Sons: New York, , 90, 1240–1241(1986).

[b30] Vidal-Abarca C., Lavela P. & Tirado J. L. The origin of capacity fading in NiFe_2_O_4_ conversion electrodes for lithium ion batteries unfolded by 57Fe Mossbauer spectroscopy. J. Phys. Chem. C 114, 12828–12832 (2010).

[b31] Wang N. . A general approach for MFe_2_O_4_ (M= Zn, Co, Ni) nanorods and their high performance as anode materials for lithium ion batteries. J. Power Sources. 247, 163–169 (2014).

[b32] Cherian C. T. . Morphologically robust NiFe_2_O_4_ nanofibers as high capacity Li-ion battery anode material. ACS Appl. Mater. Interfaces. 5, 9957–9963 (2013). 24099146 10.1021/am401779p

[b33] Zhang G. & Lou X. W. D. General Solution Growth of Mesoporous NiCo_2_O_4_ Nanosheets on Various Conductive Substrates as High-Performance Electrodes for Supercapacitors. Adv. Mater. 25, 976–979 (2013). 23225205 10.1002/adma.201204128

[b34] Jiang H., Ma J. & Li C. Hierarchical porous NiCo_2_O_4_ nanowires for high-rate supercapacitors. Chem. Commun. 48, 4465–4467 (2012). 10.1039/c2cc31418e 22453815

[b35] Yuan C. . X. Facile template-free synthesis of ultralayered mesoporous nickel cobaltite nanowires towards high-performance electrochemical capacitors. J. Mater. Chem. 22, 16084–16090 (2012).

[b36] Li L. . Electrospun porous NiCo_2_O_4_ nanotubes as advanced electrodes for electrochemical capacitors. Chem.–Eur. J. 19, 5892–5898 (2013). 23494864 10.1002/chem.201204153

